# Improving the prediction performance of leaf water content by coupling multi-source data with machine learning in rice (*Oryza sativa* L.)

**DOI:** 10.1186/s13007-024-01168-5

**Published:** 2024-03-23

**Authors:** Xuenan Zhang, Haocong Xu, Yehong She, Chao Hu, Tiezhong Zhu, Lele Wang, Liquan Wu, Cuicui You, Jian Ke, Qiangqiang Zhang, Haibing He

**Affiliations:** 1 Agricultural College, Anhui Agricultural University, Hefei, 230036 Anhui People’s Republic of China; 2 Collaborative Innovation Center for Modern Crop Production Co-Sponsored by Province and Ministry (CIC-MCP), Nanjing, 210095 Jiangsu People’s Republic of China; 3 Yingshang Agricultural Green Development Promotion Center, Fuyang, 236200 Anhui People’s Republic of China; 4 Germplasm Creation and Application Laboratory of Grain and Oil Crops in Wanjiang Plain, Enterprise Key Laboratory of Ministry of Agriculture and Rural Affairs, Tongling, 244002 China

**Keywords:** Rice (*Oryza sativa* L.), Leaf water content, Hyperspectral remote sensing, Machine learning

## Abstract

**Background:**

Leaf water content (LWC) significantly affects rice growth and development. Real-time monitoring of rice leaf water status is essential to obtain high yield and water use efficiency of rice plants with precise irrigation regimes in rice fields. Hyperspectral remote sensing technology is widely used in monitoring crop water status because of its rapid, nondestructive, and real-time characteristics. Recently, multi-source data have been attempted to integrate into a monitored model of crop water status based on spectral indices. However, there are fewer studies using spectral index model coupled with multi-source data for monitoring LWC in rice plants. Therefore, 2-year field experiments were conducted with three irrigation regimes using four rice cultivars in this study. The multi-source data, including canopy ecological factors and physiological parameters, were incorporated into the vegetation index to accurately predict LWC in rice plants.

**Results:**

The results presented that the model accuracy of rice LWC estimation after combining data from multiple sources improved by 6–44% compared to the accuracy of a single spectral index normalized difference index (ND). Additionally, the optimal prediction accuracy of rice LWC was produced using a machine algorithm of gradient boosted decision tree (GBDT) based on the combination of ND_(1287,1673)_ and crop water stress index (CWSI) (R^2^ = 0.86, RMSE = 0.01).

**Conclusions:**

The machine learning estimation model constructed based on multi-source data fully utilizes the spectral information and considers the environmental changes in the crop canopy after introducing multi-source data parameters, thus improving the performance of spectral technology for monitoring rice LWC. The findings may be helpful to the water status diagnosis and accurate irrigation management of rice plants.

**Supplementary Information:**

The online version contains supplementary material available at 10.1186/s13007-024-01168-5.

## Introduction

Rice is an important staple food worldwide. Rice is the largest water-consuming crop in the central rice production regions in the world, and water scarcity is bound to threaten rice production [1]. Additionally, the water consumption in paddy fields would increase significantly due to global climate change in the future. The unfavorable factors must aggravate the water crisis in rice fields [2, 3]. Leaf water content (LWC) is an important evaluation index for crop water demand status [4]. Monitoring LWC effectively achieves precise irrigation, improves water utilization, and alleviates the water crisis.

Hyperspectral remote sensing technology has recently been widely used in agricultural production due to its rapid, nondestructive, and real-time monitoring of crop physiological and biochemical characteristics [5–7]. Early LWC spectral modeling studies improved prediction accuracy by constructing various types of vegetation indices from sensitive spectral bands, such as the moisture stress index (MSI) and water index (WI) [8, 9]. Previous spectral monitoring studies have initially focused on vegetation indices derived from spectral bands. Researchers construct different vegetation indices by spectrally sensitive bands to improve the prediction accuracy of LWC [10, 11]. Vegetation indices are frequently screened by selecting spectral information and have a good correlation with LWC at multiple points over many years [12, 13], but the established models usually ignore the effects of the growing environment and growth characteristics on LWC of the crops. Climate characteristics and physiological status of the plants are important factors affecting LWC [14, 15]. The neglected information could be the primary reason for the poor generalizability of existing vegetation index models in practical applications. Therefore, integrating the actors into the spectral monitoring models of LWC would be more high precision due to considering the potential effects of the growing environment and growth characteristics on LWC [16, 17]. Qin et al. [18] have recently improved crop nitrogen content prediction accuracy using a spectral model that combines image feature parameters and fluorescence parameters. These results supported our inferences that introducing multi-source data may be important means to improve the monitoring accuracy of LWC spectral modeling in rice plants.

Choosing modeling methods are one of key steps in building monitoring models. Vegetation index monitoring models have been usually established using linear and nonlinear functions [19, 20]. These traditional monitoring models do not represent the complex relationship between various indicators. Machine learning algorithms with sophisticated functionality and the ability to handle complex relationships between predictors and target variables can be a good solution to this problem [21, 22]. The accuracy and stability of models have significantly improved with the rapid development of machine learning algorithms, and these are widely adopted when establishing rice nitrogen nutrition monitoring models [23–25]. Moreover, machine learning algorithms allow the use of different classes of sample data as input variables, allowing multiple sources of data (physiological and ecological indicators and spectral information) to be effectively coupled and can effectively discriminate between differences in the contributions of the input variables, allowing the model’s parameters can be fully utilized. Therefore, we speculated that the monitoring capacities of vegetation index models integrating with multi-source data would also have significant advantages using machine learning algorithms when predicting the LWC of rice plants based on the results previous studies [16, 26].

Three irrigation regimes and four varieties with different drought tolerance capacities were established LWC difference populations in field experiments. This study’s objectives are to (1) select spectrally sensitive bands with high correlation with LWC at multiple growth stages; (2) build a new vegetation index model of LWC integrating multi-source data parameters after selecting key physiological and ecological indicators; and (3) use a machine learning algorithm to optimize the coupling model and select the optimal algorithm to monitor the LWC.

## Materials and methods

### Site and treatment description

**Fig. 1:**
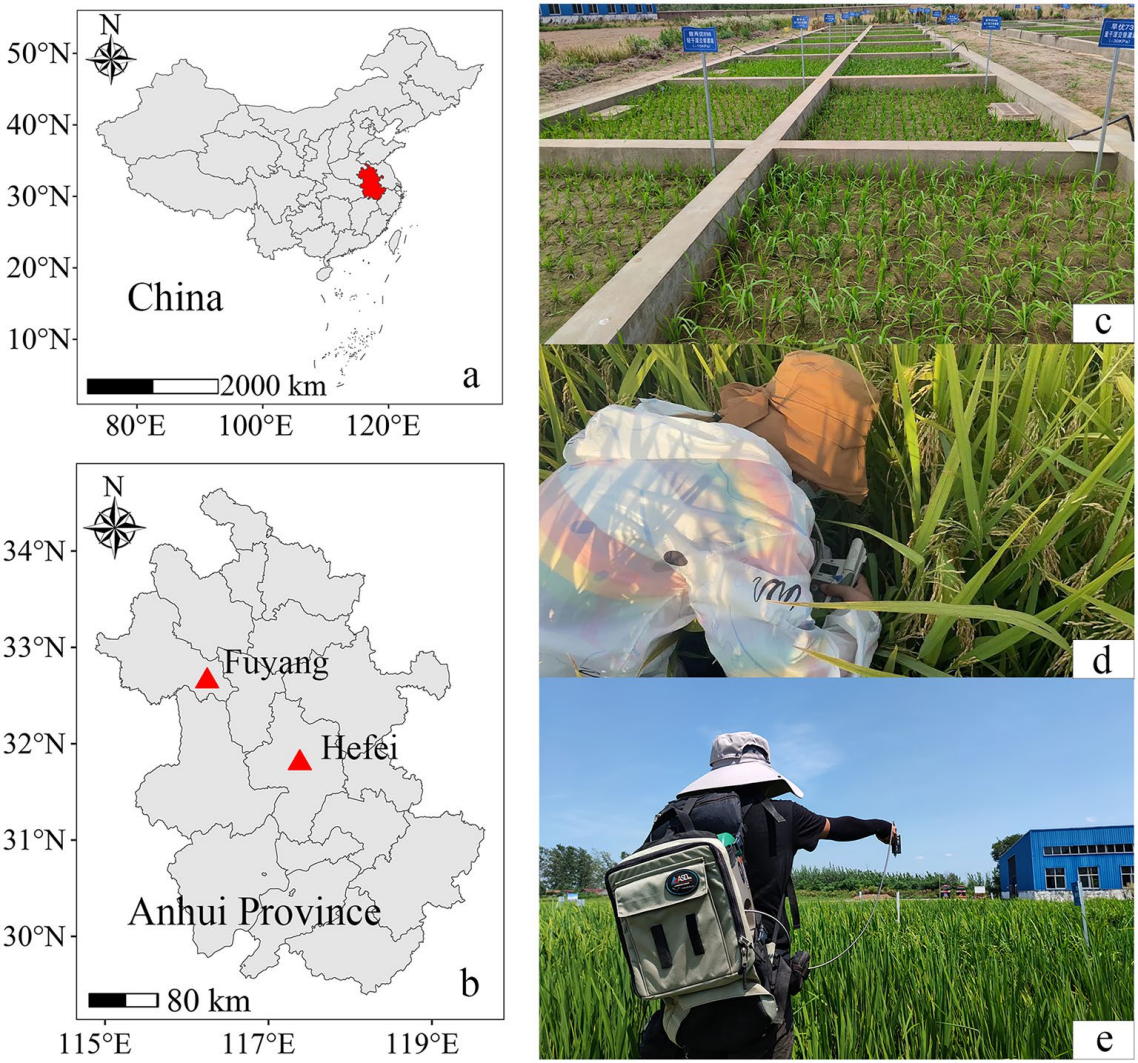
Sketch of field experiments (**c**) in Anhui province (**b**), China (**a**) using ASD Field Spec 4 (**e**) and SPAD (**d**) devices

**Fig. 2:**
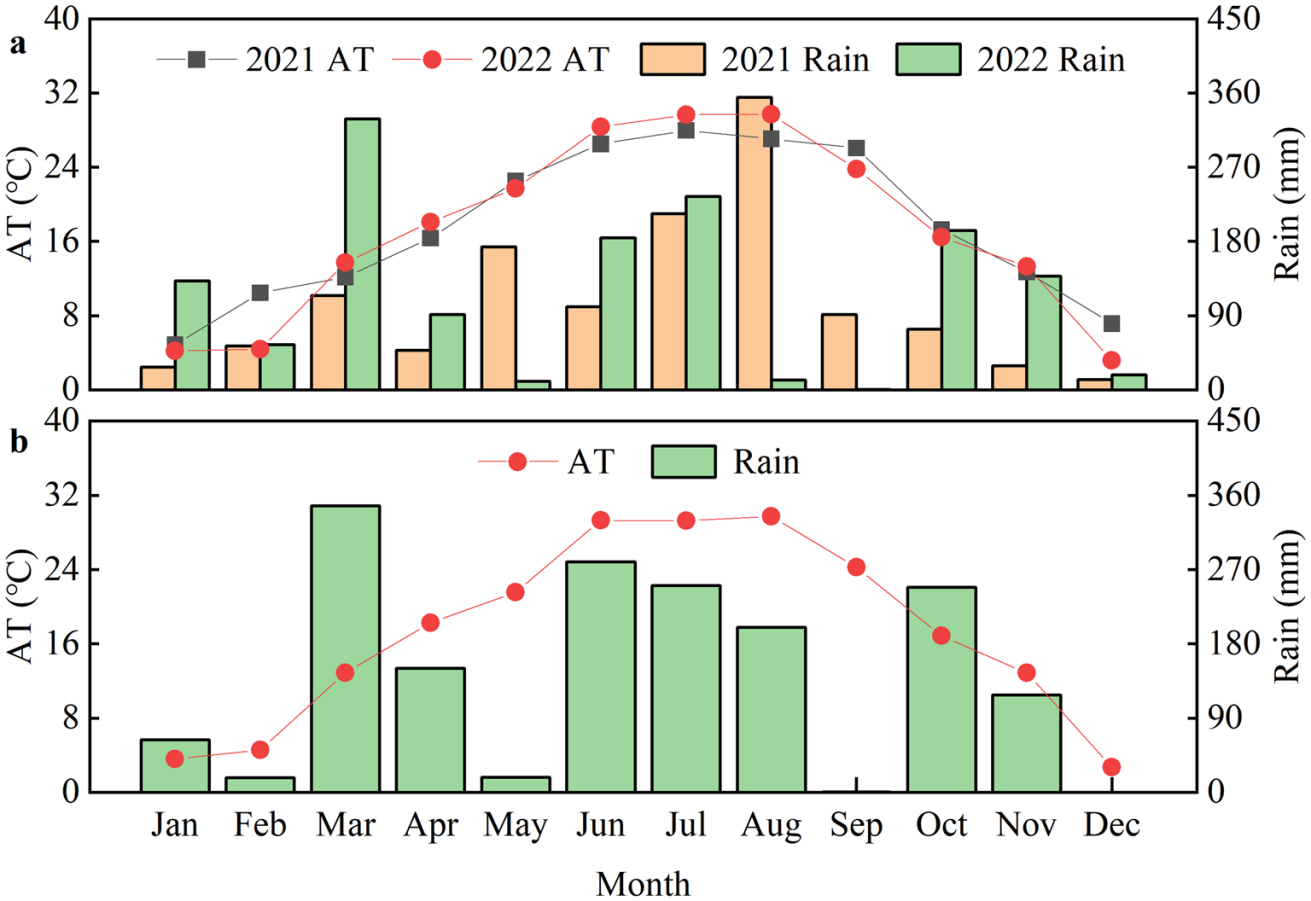
Climatic conditions at Wanzhong Comprehensive Experimental Station during the 2021 and 2022 seasons (**a**). Climatic conditions at Yingshang Agricultural Green Development Experiment Station during 2022 (**b**). AT: monthly average temperature; Rain: monthly accumulated rainfall

Three irrigation regimes, including two water-saving irrigation regimes named mild alternate dry and wet irrigation (MADW), severe alternate dry and wet irrigation (SADW) and traditional irrigation regime (CK), were conducted with four rice cultivars in Anhui province of China in 2021 and 2022 (Fig. 1). The planting and sampling plan details and soil characteristics are presented in Table 1. The climate conditions are displayed in Fig. 2. The irrigation regimes were conducted when rice seedlings were planted in plots. The irrigation criterion referenced the classical definition for the MADW and SADW treatments [27]. The supplementary irrigation with 2–3 cm water layer was carried out when the soil water potential at 20 cm soil layer reached − 15 KPa and − 30 KPa in the MADW and SADW treatments during whole growth stages of rice plants, respectively, across cultivars, years, and locations. The soil water potential of each plot was monitored by a tensiometer (Watermark, Irrometer Company Riverside, CA, USA). Moreover, 2–3 cm water later was always maintained for the CK treatment during whole growth stages. Generally, 9–14, 5–8, and 18–24 times irrigated practices were applied for the MADW, SADW, and CK treatments, respectively, across cultivars, years, and locations.

**Table 1:** Basic information of the three experiments

Experiment	Year	Cultivar	Soil water potential	Sampling stage	Sowing/Harvesting date	Soil characteristics
Exp.1 (Hefei, 31° 48′ N, 117° 23′ E)	2021	Hanyou 73 (HY-73) Huanghuazhan (HHZ) Huaidao 5 (HD-5)	0 KPa − 15 KPa − 30 KPa	Booting Flowering Initial grain filling Middle grain filling	July 5th / November 20th	Type: sandy loam soil Organic matter: 14.81 g kg^−1^ Total N: 0.97 g kg^−1^ Available P: 21.06 mg kg^−1^ Available K: 96.29 mg kg^−1^
Exp.2 (Hefei, 31° 48′ N, 117° 23′ E)	2022	Hanyou 73 (HY-73) Huanghuazhan (HHZ) Huiliangyou 898 (HLY-898)	0 KPa − 15 KPa − 30 KPa	Booting Flowering Initial grain filling Middle grain filling	May 23th/ September 26th	Type: sandy loam soil Organic matter: 15.39 g kg^−1^ Total N: 1.00 g kg^−1^ Available P: 22.67 mg kg^−1^ Available K: 98.10 mg kg^−1^
Exp.3 (Fuyang, 32° 39′ N, 116° 15′ E)	2022	Hanyou 73 (HY-73) Huanghuazhan (HHZ)	0 KPa	Booting Flowering Initial grain filling Middle grain filling	May 26th/ September 29th	Type: sandy loam soil Organic matter: 17.77 g kg^−1^ Total N: 0.95 g kg^−1^ Available P: 14.18 mg kg^−1^ Available K: 65.23 mg kg^−1^

The nitrogen fertilizer was divided into three applications for each treatment: 40% as a basal fertilizer, 30% at the early tiller stage, and 30% at the panicle differentiation stage. The nitrogen fertilizer was applied at the rate of 240 kg ha^−1^. Phosphorus and potassium fertilizers (P_2_O_5_ 75 kg ha^−1^ and K_2_O 225 kg ha^−1^) were also applied as basal fertilizers. Plot sizes for each experiment were 12 m^2^ and 40 m^2^ in Hefei and Fuyang, respectively. All experimental rows were spaced 30.0 cm apart, and plants in a row were spaced 13.3 cm apart.

### Measurements

#### Hyperspectral and vegetation index

Canopy reflectance spectra of rice plants were collected for the booting, flowering, initial grain filling, and middle grain filling stages using spectral scanning equipment (ASD Field Spec 4, Boulder, CO, USA). The band amplitude of the device ranged from 350 to 2500 nm. The measurements were conducted under clear and cloudless sky conditions between 10:00 and 14:00 [16]. After measuring 10 times for each sample, the average value was calculated, and the reference plate was used to correct the instrument every 15 min. The representative hills were monitored in each plot for all treatments at each observed time.

Numerous vegetation indices have been applied to monitoring crop water content. The normalized difference vegetation index (NDVI) has been widely utilized in monitoring plant water using remote sensing methods due to its simple construction and effective improvement of spectral monitoring accuracy with multi-band analysis compared with other vegetation indices. Thus, this study used canopy spectral data to construct the normalized difference index (ND) and five traditional vegetation indices. The calculation equations were illustrated in Table 2.

**Table 2:** List of vegetation indices used in this study

Vegetation index	Algorithm	Reference
Normalized difference index (ND)	ND = (R_λ1_ − R_λ2_)/ (R_λ1_ + R_λ2_)	This study
Normalized difference vegetation index (NDVI)	NDVI = (R_895_ − R_675_)/(R_895_ + R_675_)	[28]
Normalized difference infrared index (NDII)	NDII = (R_819_ − R_1600_)/(R_819_ + R_1600_)	[29]
Normalized difference water index (NDWI)	NDWI = (R_860_ − R_1240_)/(R_860_ + R_1240_)	[30]
Water index (WI)	WI = R_970_/R_900_	[9]
Moisture stress index (MSI)	MSI = R_1600_/R_820_	[8]

The λ1 and λ2 are arbitrary bands in the 350–2500 nm bands, and R_λ1_ and R_λ2_ are the spectral reflectance of the bands

#### SPAD value and chlorophyll fluorescence parameters

SPAD value of rice leaves was monitored using a chlorophyll meter (Konica Minolta Company, SPAD-520 plus, Japan). The average value of the top three fully expanded leaves was defined as the SPAD value of the whole plant. The five independent plants were measured in each plot across cultivars, water treatments, and observed periods.

The leaves used to measure SPAD value were chosen, and chlorophyll fluorescence parameters of rice leaves were determined using a portable pulse-modulated chlorophyll fluorometer (WALZ Company, PAM-2500, Germany). These leaves were used to determine the minimum fluorescence level (*F*o) and the maximum fluorescence level (*F*m) of dark-adapted leaves at dawn for all cultivars and treatments. The steady-state *F*o was measured using far-red light with illumination less than 1 mol m^−2^ s^−1^. Then, a 0.80-s saturating pulse with 8000 mol m^−2^ s^−1^ PAR was supplied to determine the *F*m. Moreover, the steady-state fluorescence yield (*F*s) was recorded at the forenoon with clear and cloudless sky conditions. Then, a 0.80-s saturating pulse with 8000 mol m^−2^ s^−1^ PAR was stimulated to obtain the maximal fluorescence level (*F*m'). Finally, the maximum photochemical efficiency (*F*v/*F*m) and actual photochemical efficiency (Y(II)) were calculated:

1 $${\text{Fv}}/Fm = (Fm - Fo)/Fm$$

2 $${\text{Y(II)}} = (Fm^{\prime} - Fs)/Fm^{\prime}$$

#### Leaf water content (LWC)

The fresh leaf mass (FW) was determined by weighing the top three fully expanded leaves immediately. Five hills were sampled in each plot for four cultivars and three water treatments during each observed period. Fresh leaves were weighed and dried at 80 ℃ to constant weight at each sampling. The weight was marked as dry mass (DW). The LWC was calculated as follows:

3 $${\text{LWC}} = (FW - DW)/FW$$

#### Crop water stress index (CWSI)

CWSI was calculated as follows [31, 32]:

4 $${\text{CWSI}} = \frac{(Tc - Ta) - T\min }{{T\max - T\min }}$$

5 $${\text{Tmin}} = A + B \times VPD$$

6 $${\text{Tmax}} = A + B \times VPG$$

7 $${\text{VPD}} = 0.61 \times e^{{\frac{17.27Ta}{{Ta + 237.3}}}} \times (1 - \frac{RH}{{100}})$$

where Tc is the crop canopy temperature (℃), and Ta is the air temperature (℃). Tmin is the lower limit of the canopy and air temperature difference (℃), Tmax is the upper limit of the canopy and air temperature difference (℃), and VPD is the air saturation water vapor pressure deficit (KPa). VPG is the difference between the air saturation water vapor pressure and VPD when the air temperature is Ta and (Ta + A), respectively. A and B are linear regression coefficients. RH is the relative humidity of air (%).

In this study, a HOBO UX100-003 temperature and humidity recorder (Onset, USA) was placed in each plot to automatically record rice canopy temperature and humidity every 1 h interval throughout the day.

#### Leaf area index (LAI), above-ground biomass (Biomass), and Grain yield

Five representative hills, which plants in the hills had uniform growth capacities, were randomly selected from each plot at each sampling time across all cultivars and irrigation treatments. The hills with excessively vigorous or weak plants were not sampled to minimize sampling error. All green leaves per hill were scanned using a portable leaf area meter (CI-203, CID Inc., USA). The averaged leaf area in a each plot was then calculated from the five hills and marked with D (cm^2^). Finally, the scanned green leaf, remaining yellow leaves, stem, and panicle organs were dried at 80 ℃ to constant weight to assess the biomass of each plot.

8 $${\text{LAI}} = \frac{D \times \rho }{{10000}}$$

where ρ is the planting density per square meter (hill m^−2^).

Plants from a 2 m^2^ area were harvested at maturity to calculate the actual grain yield with 13.5% moisture content in each plot across cultivars and water treatments.

### Regression models

Some common and classic machine learning algorithms were preliminary assessed to select elite algorithms with high monitoring abilities to LWC of rice plants. Finally, the four machine learning algorithms named decision tree regression (DT), random forest regression (RF), K-nearest neighbor regression (KNN), and gradient boosting decision tree regression (GBDT) were adopted in this study. The four selected methods were successfully applied on estimating various ecological parameters such as water content of wheat plants and soil moisture of wheat fields [16, 33]. In addition, the scikit-learn packages of the four algorithms were from Python 3.8 software (https://scikit-learn.org/stable/index.html).

#### Multiple linear regression (MLR)

MLR is the most basic and commonly used method for combining two or more independent variables that jointly predict or estimate the dependent variable [34]. The y is the dependent variable. The x_1_, x_2_… x_k_ are the independent variables. The multiple linear regression was calculated as follows:

9 $${\text{y}} = {\text{b}}_{0} + {\text{ b}}_{{1}} {\text{x}}_{{1}} + \cdots \, + {\text{ b}}_{{\text{k}}} {\text{x}}_{{\text{k}}} + {\text{e}}$$

where b_0_ is the constant term, e is the error term, and b_1_, b_2_… b_k_ are the regression coefficients. When x_1_, x_2_…, and x_k_ are fixed, b_1(k)_ represents the effect of increase or decrease in x_1(k)_ on y for each unit or named the partial regression coefficient of x_1(k)_ on y.

#### Decision tree regression (DT)

DT is a way to infer classification rules as a decision tree from a set of unordered and irregular data, using a top-down recursive approach to compare attribute values of nodes inside the decision tree [35]. Each internal node is a splitting problem in a decision tree. A test for some instance attribute is specified, and it splits the samples arriving at that node according to a particular attribute. Each subsequent branch of the node corresponds to one of the possible values of that attribute. The prediction results are the average values of the output variables in the samples contained in the leaf nodes of the regression tree. In this study, the maximum depth of the tree was set as 10, and the number of trees was 100.

#### Random forest regression (RF)

RF resulted from random sampling from sample observations and feature variables of the modeled data among many decision trees; each sampling result is a tree [36]. Meanwhile, each tree generated rules and judgment values that match its properties. Finally, the forest algorithm integrated the rules and judgment values of all decision trees to achieve random forest regression.

#### K-nearest neighbor regression (KNN)

KNN was predicted by computing the spatial similarity relationship between the k nearest neighbors and the predictor. The algorithm was frequently used for classification problems in the early stage and gradually applied to parameter estimation [37]. The primary principle of the KNN algorithm is that a prediction sample has K nearest neighbors in the feature space. Then, the class of the prediction sample was usually determined by the majority class of the K nearest neighbors. The K data set value was chosen appropriately according to the samples. The model was simplified, and useful information was lost if the data set was too large. Oppositely, the model would be over-fitted if the data set is too small. The K data set was defined as 3 in this study.

#### Gradient boosting decision tree regression (GBDT)

GBDT is an improved algorithm based on the booting algorithm [38]. The booting algorithm assigned the same weight to each training sample in the initial training and then increased the weights of the error points after each training session to generate multiple base learners. Finally, these base learners were combined, and the model was formed using weighting or voting approaches. The difference between gradient boosting tree regression and classification algorithms was that the input training data was residual. The previous prediction was incorporated into the residual to find the training data for the current round instead of the gradient of the loss function.

### Data analysis and model verification

The Pearson correlation coefficient (PCC) helps to measure the linear correlation between variables [39]. In this study, PCC was simply regarded as supplementary means to eliminate unimportant indicators with low correlation coefficient and to obtain eigenvalues with high correlation coefficient. This meant that indicators closely related to LWC were used to build models to improve performance.

After removing invalid samples from the two-year experiments from 2021 to 2022, 91 sample data for each growth stage were obtained. In multiple linear regression (MLR), 2022 data were used for modeling (55), whereas 2021 data were used for validation (36). The measured data were randomly divided into training (70%) and testing (30%) sets in the machine learning regression algorithm. The regression model's accuracy was evaluated using the determination coefficient (R^2^) and root mean square error (RMSE). The overall model was evaluated in the graph, including linear regression and a 1:1 dash-line, to determine the relationship between the predicted and measured values. The calculation equations are presented in (9)–(10):

10 $$R^{2} = \frac{{\sum\nolimits_{{i = {1}}}^{n} {(x_{i} - \hat{x}_{i} )^{2} } }}{{\sum\nolimits_{{i = {1}}}^{n} {(x_{i} - \overline{x})^{2} } }}$$

11 $$RMSE = \sqrt {\frac{{\sum\nolimits_{{i = {1}}}^{n} {(x_{i} - \hat{x}_{i} )^{2} } }}{n}}$$

where ${x}_{i}$ is the measured value of LWC, $\overline{x }$ is the measured mean value of LWC, ${\widehat{x}}_{i}$ is the predicted value of the model, and n is the sample size. The larger the R^2^ value, the better the accuracy of the model. The RMSE reflects the degree of dispersion and deviation between the model’s predicted and true values. The smaller the value, the better the prediction of the model.

**Fig. 3:**
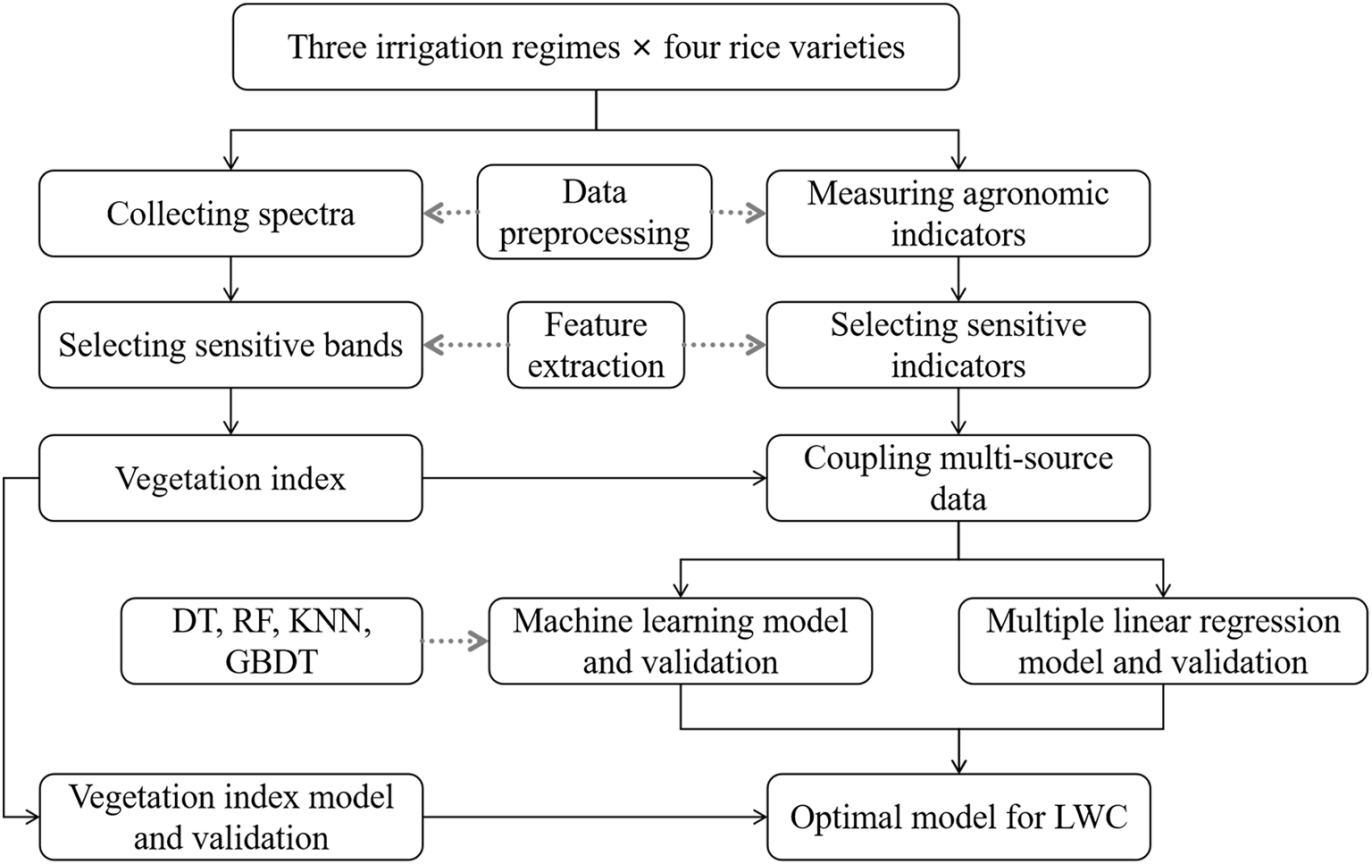
Flow chart of this study

The workflow of the LWC prediction procedures is illustrated in Fig. 3. Data preprocessing included the following processes: (1) normal distribution test and scatter plots were performed for the original data such as vegetation index parameters, leaf water content, and physiological and ecological indexes (Additional file 1: Fig. S1–S4). (2) Outliers detection of all parameters were shown in Additional file 1: Table S1. (3) The multicollinearity test between ND and SPAD, between ND and Fv/Fm, between ND and CWSI at whole observed stages were also adopted by variance inflation factor and tolerance values (Additional file 1: Table S2).

## Results

### The classical and new vegetation index for monitoring LWC

**Fig. 4:**
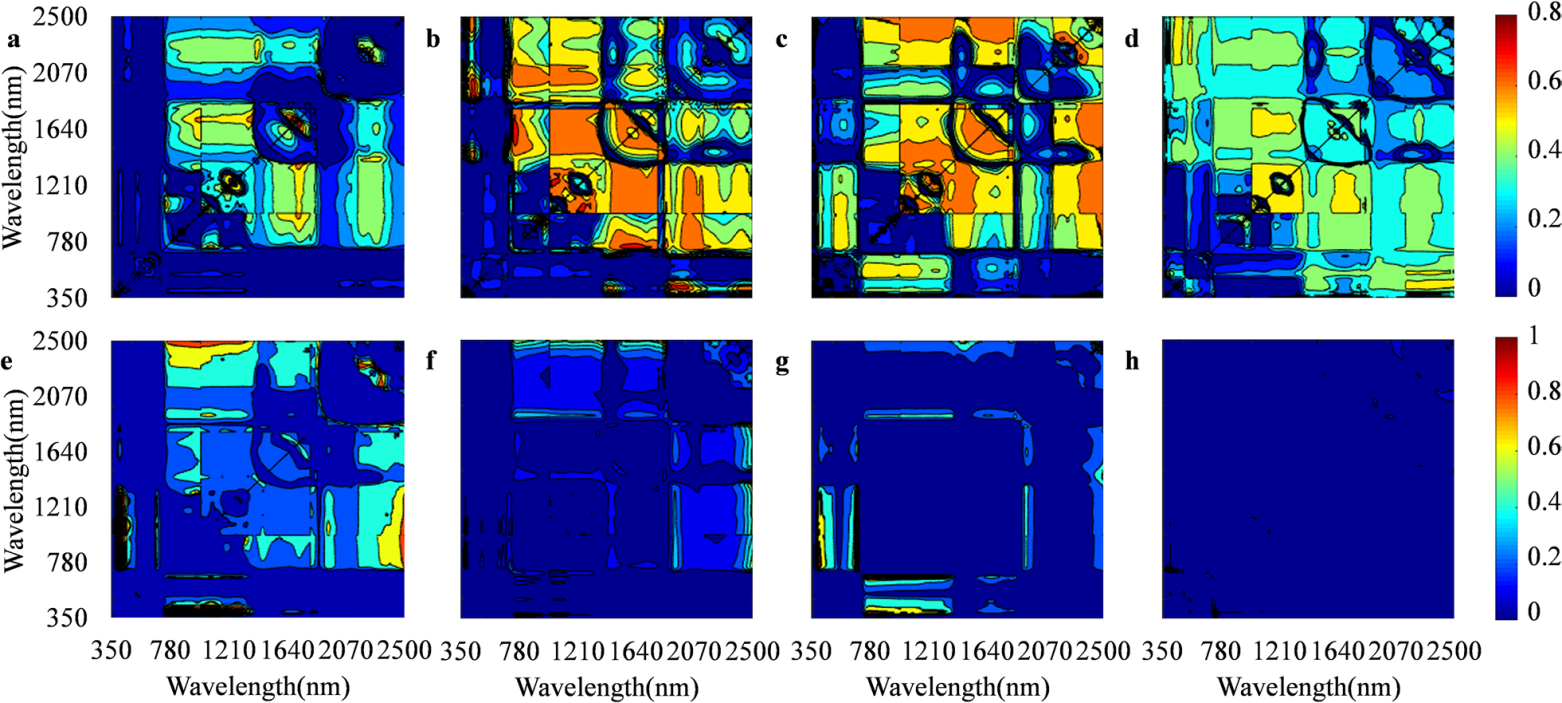
Image map of the coefficient of determination (R^2^) and coefficient of determination (RMSE) for screening sensitive band combinations of two wavebands at different growth stages of rice plants. **a**–**d** and **e**–**h** represent R^2^ and RMSE of the booting, flowering, initial grain filling, and middle grain filling stages, respectively

**Fig. 5:**
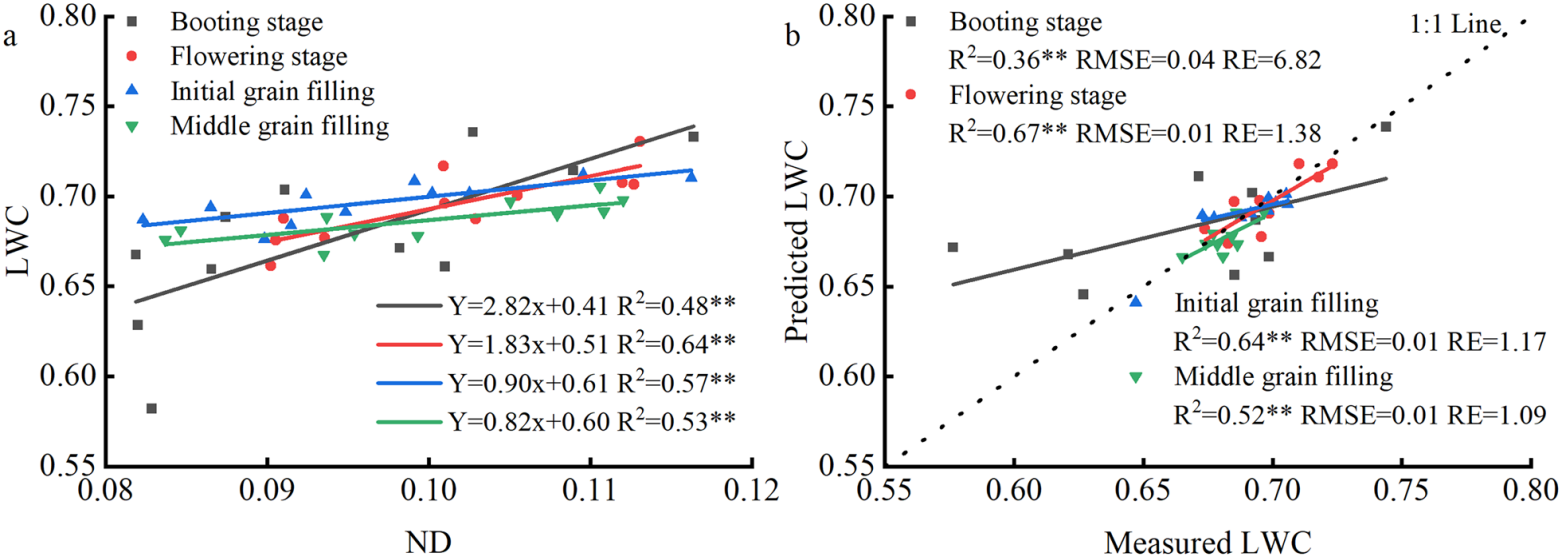
Model construction (**a**) and validation (**b**) of normalized difference index (ND) for monitoring LWC at different growth stages of rice plants. Each symbol is the average value from 5 (**a**) and 4 (**b**) repeated measurements. Dashed lines represent 1:1 lines; * and ** indicate significant correlation at 5% and 1% probability level, respectively

The sensitive bands of LWC were analyzed and tested using the normalized difference vegetation index formula at the booting, flowering, initial grain filling, and middle grain filling stages to improve the accuracy of the hyperspectral monitoring model for LWC. The classical vegetation indices, such as NDII and MSI, had a significant positive correlation with LWC and high predictive abilities (Table 3). The vegetation index ND constructed by the screened sensitive bands (1287 and 1673 mm) also had a significant positive correlation with LWC (Fig. 4). The R^2^ in the ND_(1287,1673)_ model was higher than that in NDII and MSI vegetation indices at each growth stage. These results indicated that vegetation index ND was an optimal predicted model at each growth stage of rice plants. The R^2^ of the linear equation between LWC (*y*) and ND_(1287,1673)_ (*x*) was 0.48, 0.64, 0.57, and 0.53, and the corresponding prediction R^2^ was 0.36, 0.67, 0.64, and 0.52 at the booting, flowering, initial grain filling, and middle grain filling stages, respectively (Fig. 5).

**Table 3:** Coefficient of determination (R^2^) of predicting LWC by different vegetation index at different growth stages in rice plants

Growth stage	ND_(1287,1673)_	NDVI	NDII	NDWI	WI	MSI
Booting	0.48**	0.01	0.26**	0.00	0.01	0.22**
Flowering	0.64**	0.02	0.48**	0.06	0.27**	0.47**
Initial grain filling	0.57**	0.24**	0.30**	0.03	0.09*	0.30**
Middle grain filling	0.53**	0.01	0.34**	0.32**	0.34**	0.34**

* and ** indicate significant correlation at 5% and 1% probability level, respectively. ND_(1287,1673)_ is the normalized difference index; NDVI is the normalized difference vegetation index; NDII represents the normalized difference infrared index; NDWI is the normalized difference water index; WI shows the water index; MSI represents the moisture stress index

### The relationship between physiological-ecological indicators and LWC

**Fig. 6:**
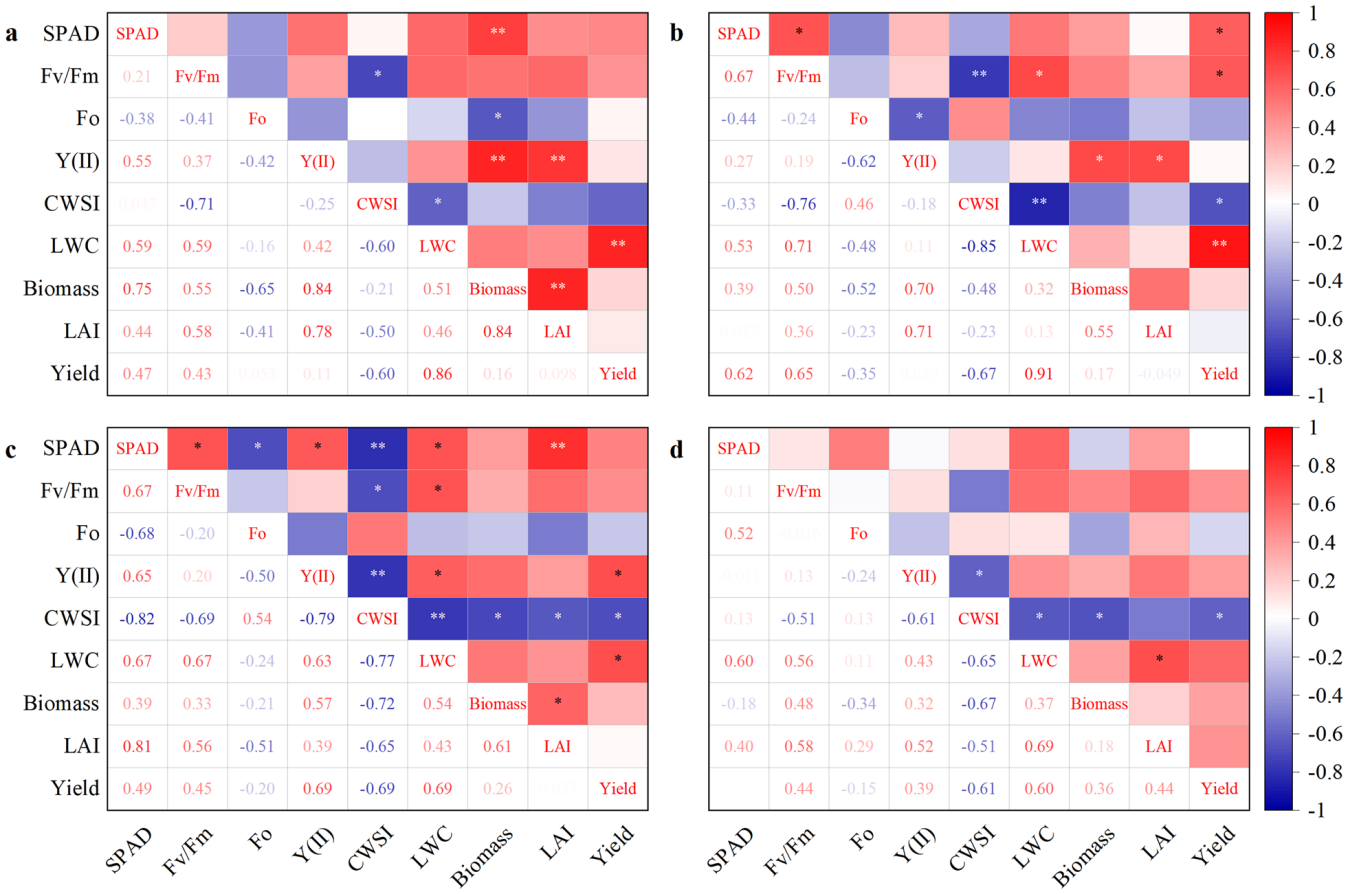
Pearson correlation coefficient (PCC) among LWC, biomass, LAI, CWSI, SPAD, *F*o, *F*v/*F*m, Y(II), and yield at different growth stages. **a**–**d** Represents the PCC of the booting, flowering, initial grain filling, and middle grain filling stages, respectively. * and ** indicate significant correlation at 5% and 1% probability level, respectively. SPAD: chlorophyll content; *F*v/*F*m: maximum photochemical efficiency; *F*o: minimal fluorescence; Y(II): actual photochemical efficiency; CWSI: crop water stress index; LWC: leaf water content; Biomass: above-ground biomass; LAI: leaf area index

The physiological-ecological indicators had obvious effects on LWC at each growth stage and yield (Fig. 6). The LWC had the highest correlation with grain yield at the booting, flowering, and initial grain filling stages. The LWC is the most important parameter that regulates grain yield, especially for different irrigation treatments. CWSI had the highest correlation coefficient with LWC (− 0.60 to − 0.85) at each observed stage, followed by the *F*v/*F*m (0.56–0.71) and SPAD (0.53–0.67). Therefore, CWSI, *F*v/*F*m, and SPAD can be considered reliable multi-source data to improve the detection accuracy of LWC using the vegetation index model.

**Fig. 7:**
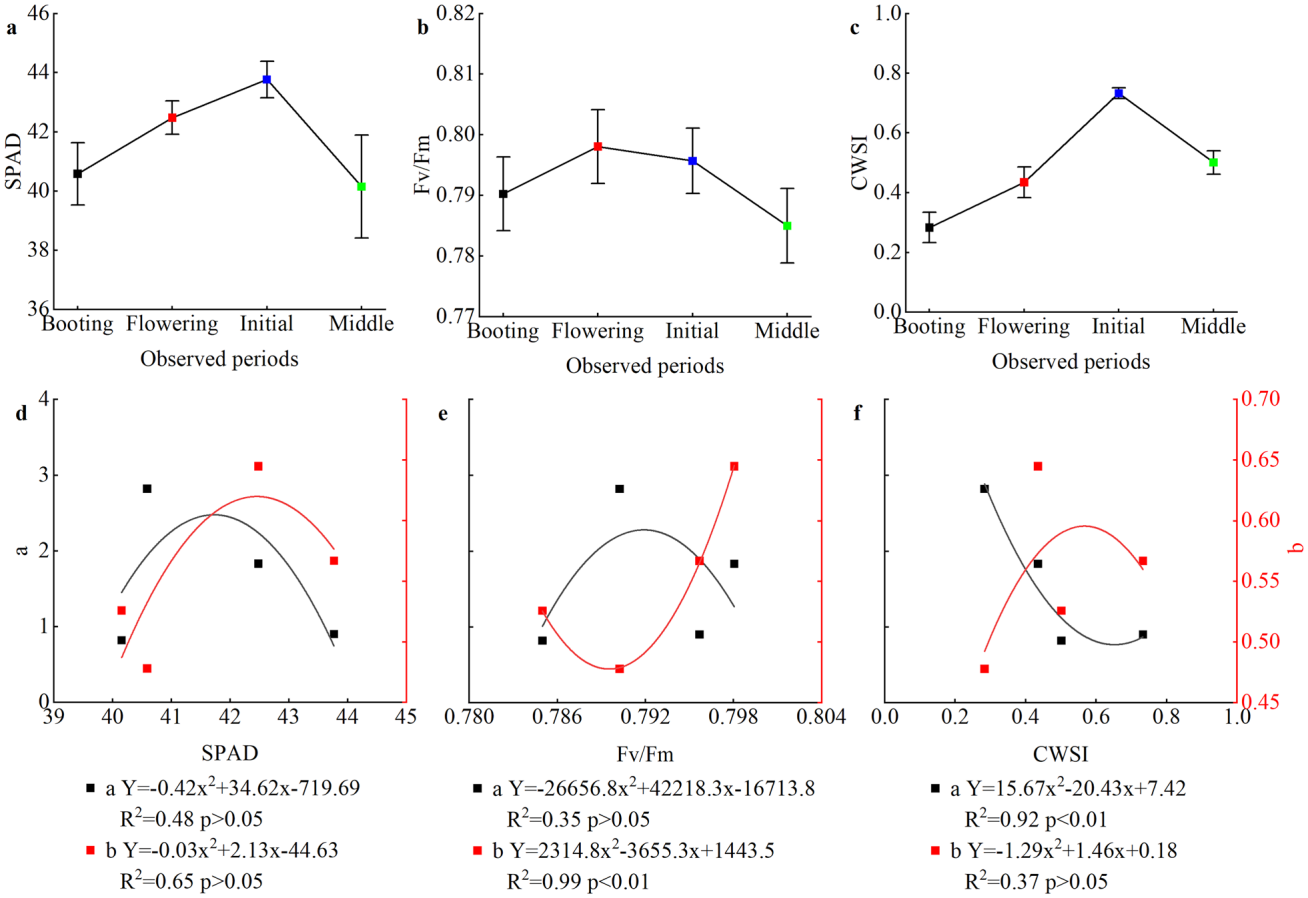
The SPAD value (**a**), *F*v/*F*m (**b**), and CWSI (**c**) at different growth stages merged cultivars, water treatments, and years. The quadratic function relations of both a (black line) and b (red line) parameters derived from the ND_(1287,1673)_ model in Fig. 4a with SPAD **d**, *F*v/*F*m (**e**), and CWSI (**f**). Booting: booting stage; Flowering: flowering stage; Initial: initial grain filling; Middle: middle grain filling; p < 0.05 and p < 0.01 indicate significant correlation at 5% and 1% probability level, respectively

In this study, the slope (a) and intercept (b) of linear functions between LWC and ND_(1287,1673)_ at different growth stages (Fig. 5) were systematically analyzed using multi-source data, such as SPAD, *F*v/*F*m, and CWSI (Fig. 7). Both a and b have significantly quadratic curve relations with CWSI, SPAD, and Fv/Fm. For a parameter, the determination coefficient (R^2^) of the curves reached the highest for the CWSI (R^2^ = 0.92), followed by the SPAD and *F*v/*F*m. In contrast, *F*v/*F*m had the greatest R^2^ with parameter b (R^2^ = 0.99), while CWSI had the lowest R^2^ with parameter b (R^2^ = 0.37). The results illustrate that SPAD, *F*v/*F*m, and CWSI can be regarded as useful multi-source data to improve the prediction capacities of LWC.

### Improving the prediction performance of LWC by coupling multi-source data

**Fig. 8:**
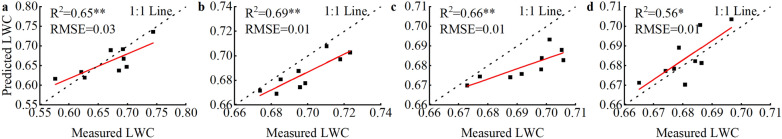
The 1:1 validation of the ND_(1287,1673)_ and multi-source data coupling model for monitoring LWC at different growth stages. **a**, **b** represents ND + CWSI model validation of the booting and flowering stages, and **c**, **d** represents ND + *F*v/*F*m model validation of the initial and middle grain filling stages. Each symbol is the average value of 4 measurements. * and ** indicate significant correlation at 5% and 1% probability level, respectively

There were no multicollinearity between ND and SPAD, between ND and *F*v/*F*m, between ND and CWSI based on the tolerance and variance inflation factor values (Additional file 1: Table S2). Also, Durbin Watson test indicated that the established multivariate linear regressed models in this study were basically meet the requirements of linear model (Additional file 1: Table S2). The indicators CWSI, *F*v/*F*m, and SPAD were introduced into the ND_(1287,1673)_ model to improve the prediction model accuracy of LWC further (Table 4). The results demonstrated that the ND_(1287,1673)_ model integrating with multi-source data enhanced predictive power than the conventional ND_(1287,1673)_ model (Fig. 5 and Table 4). The coupled models of ND_(1287,1673)_ and CWSI had the best monitoring capacity to LWC at the booting and flowering stages (Fig. 8). The R^2^ improved from 0.48–0.64 in conventional ND_(1287,1673)_ models to 0.69–0.75 in the coupled models. Prediction R^2^ also improved from 0.36–0.67 in conventional ND_(1287,1673)_ models to 0.65–0.69 in the coupled models at the booting and flowering stages. Moreover, the coupling models of ND_(1287,1673)_ and *F*v/*F*m were optimal at the initial and middle grain filling. The R^2^ and the corresponding prediction R^2^ improved in the coupling models than in conventional ND_(1287,1673)_ models at the initial and middle grain filling.

**Table 4:** The ND_(1287,1673)_ models predicting LWC after integrating with multi-source data

Growth stage	Model	Multiple linear regression equation	Model Precision	Prediction Precision	RMSE
Booting	ND + SPAD	LWC = 1.91ND + 0.02SPAD − 0.19	0.60*	0.53*	0.04
Booting	ND + *F*v/*F*m	LWC = 2.75ND + 1.99*F*v/*F*m − 1.16	0.61*	0.62**	0.03
Booting	ND + CWSI	LWC = 1.22ND − 0.34CWSI + 0.66	0.69**	0.65**	0.03
Flowering	ND + SPAD	LWC = 1.48ND + 0.01SPAD + 0.19	0.71**	0.60*	0.02
Flowering	ND + *F*v/*F*m	LWC = 2.10ND + 0.37*F*v/*F*m + 0.18	0.74**	0.64*	0.02
Flowering	ND + CWSI	LWC = 1.49ND − 0.08CWSI + 0.57	0.75**	0.69**	0.01
Initial grain filling	ND + SPAD	LWC = 1.15ND + 0.01SPAD + 0.16	0.67**	0.64**	0.01
Initial grain filling	ND + *F*v/*F*m	LWC = 1.58ND + 0.88*F*v/*F*m − 0.16	0.68**	0.66**	0.01
Initial grain filling	ND + CWSI	LWC = 1.11ND − 0.20CWSI + 0.73	0.61*	0.55*	0.01
Middle grain filling	ND + SPAD	LWC = 0.60ND + 0.002SPAD + 0.56	0.56*	0.49*	0.01
Middle grain filling	ND + *F*v/*F*m	LWC = 0.70ND + 1.19*F*v/*F*m − 0.31	0.59*	0.56*	0.01
Middle grain filling	ND + CWSI	LWC = 0.59ND − 0.10CWSI + 0.67	0.57*	0.44	0.01

LWC: leaf water content; ND_(1287,1673)_: normalized difference index; SPAD: chlorophyll content; *F*v/*F*m: maximum photochemical efficiency; CWSI: crop water stress index; * and ** indicate significant correlation at 5% and 1% probability level, respectively

### Machine learning algorithm optimization model

**Fig. 9:**
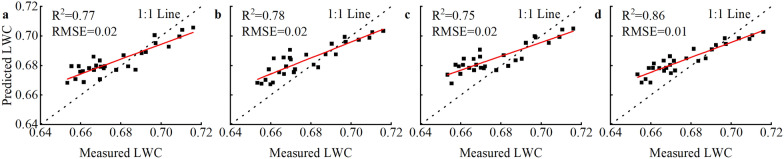
The 1:1 validation of the machine learning algorithms with ND + CWSI model for monitoring LWC at the flowering stage. **a** Decision tree regression (DT); **b** Random forest regression (RF); **c** K-nearest neighbor regression (KNN); **d** Gradient boosting decision tree regression (GBDT)

The data from the flowering stage, most sensitive to water treatments, were used as an example to assess the effects of the machine learning algorithm on ND_(1287,1673)_ combined with CWSI model precision (Table 5; Fig. 9). The R^2^ were 0.77, 0.78, 0.75, and 0.86 based on DT, RF, KNN, and GBDT models, respectively. Additionally, the RMSE was 0.01 and 0.02 for the simulating and verifying data sets in the machine learning algorithm, respectively.

**Table 5:** The R^2^ and RMSE of simulating and verifying data sets for the four machine learning algorithms

Algorithm	Simulating data set		Verification data set	
	R_S_^2^	RMSE_S_	R_V_^2^	RMSE_V_
DT	0.80	0.01	0.77	0.02
RF	0.85	0.01	0.78	0.02
KNN	0.80	0.01	0.75	0.02
GBDT	0.93	0.01	0.86	0.01

R_S_^2^: determination coefficient of simulating data set; RMSE_S_: root mean square error of simulating data set; R_V_^2^: determination coefficient of verification data set; RMSE_V_: root mean square error of verification data set

**Fig. 10:**
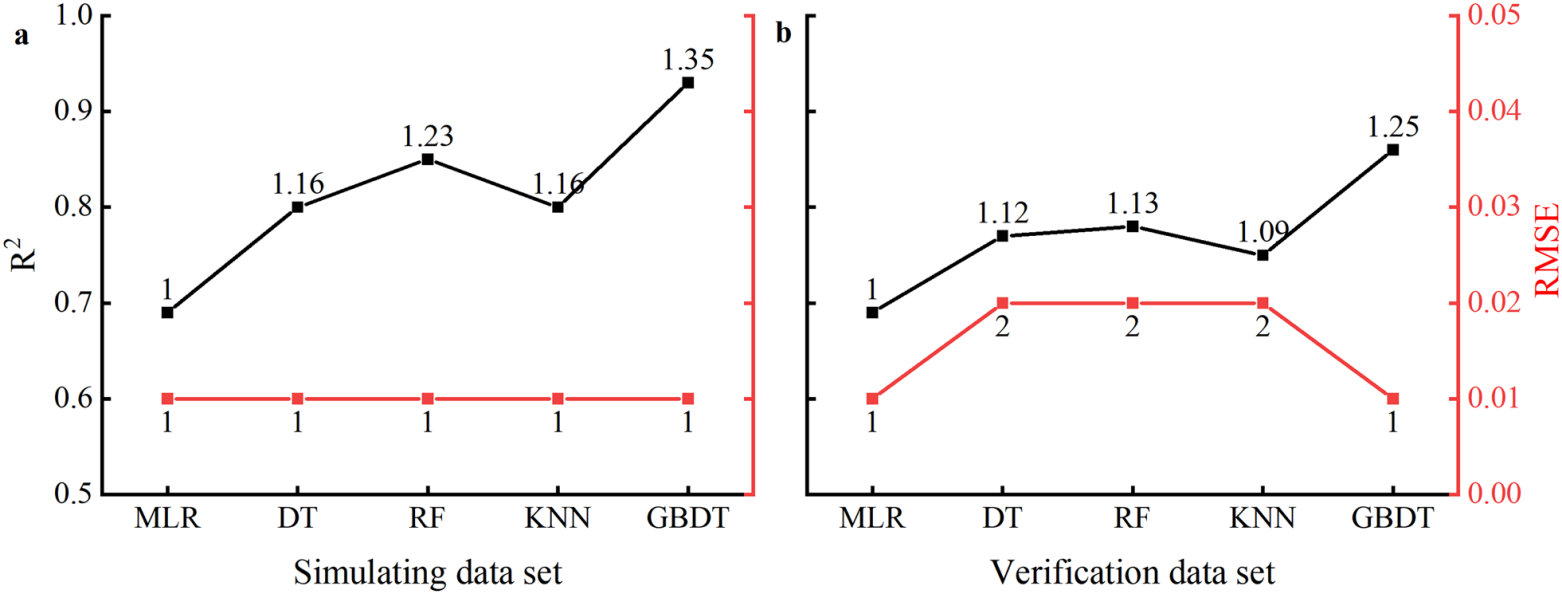
The R^2^ (black line) and RMSE (red line) of simulating (**a**) and verifying (**b**) data sets for the four machine learning algorithms and multiple linear regression. The numbers closed to the dots represent the changed folds in machine learning algorithms when compared with the normalized multiple linear regression, respectively

The R^2^ and RMSE of simulation and verification sets of machine learning algorithm were compared with multiple linear regression (MLR) model (Fig. 10). R^2^ of DT, RF, KNN and GBDT simulation sets were 1.16, 1.23, 1.16 and 1.35 times higher than that of MLR, respectively (Fig. 10). However, the RMSE of all simulated models were almost the same, which the RMSE in simulation sets were no advantage in machine learning algorithms compared with MLR. In addition, R^2^ of DT, RF, KNN and GBDT verification sets were 1.12, 1.13, 1.09 and 1.25 times higher than that of MLR, respectively. The RMSE in verification sets of DT, RF and KNN was about 2 times higher than that of MLR, but the RMSE of GBDT was at the same level when compared with MLR. Particularly, GBDT synergistically improved the R^2^ of simulating and verifying models with low RMSR level compared with MLR.

## Discussion

### Relationship between multi-source data and LWC

A suitable vegetation index is important to construct an LWC spectral monitoring model in smart agriculture. The first important step is to screen the sensitive band. However, there are some differences on the selected water-sensitive bands for different crops. Thomas et al. [40] discovered a significant correlation between the relative water content of cotton leaves and the reflectance values in the near-infrared (NIR) bands (1450 and 1930 nm). Yang et al. [41] suggest that the sensitive band range of LWC in wheat plants is located in the visible (400–780 nm) and NIR bands (1400–2500 nm). This study selected different spectral indices from previous studies to establish relationships with rice LWC. However, the correlation was not optimal. The ND constructed by the NIR band at 1287 and 1673 nm was instead optimal (Table 3). This study’s results were inconsistent with those of previous studies. One of the important reasons could be that vegetation index models for monitoring LWC in our study considered the multiple growth stages of rice plants, rather than focusing on a specific growth stage in previous studies. Additionally, the spectral data obtained from different ecological sites, climates, and varieties. In this study, the varieties were selected for their commonality bands, considering the universality of the varieties from which the four rice varieties with a large span of validation years and large differences in drought resistance were selected.

Recently, multi-source data related to environmental factors coupled with the spectral monitoring model of LWC has been successfully established in wheat plants to reduce the errors caused by environmental and physiological factors during the spectral monitoring process [16]. This study used the Pearson correlation coefficient method to analyze the physiological and ecological indicators related to LWC. Our results displayed that CWSI, *F*v/*F*m and SPAD would be considered as optimized multi-source data because the parameters had a higher correlation with LWC compared to other physiological and agronomic indices during whole observed periods (Fig. 6). These findings were also consistent with the basic physiological laws of plants that the three parameters would be changed with the change of LWC. First, the transpiration rate quickly reduces with declining LWC in rice plants [42, 43]. A low transpiration rate increases canopy temperature for a short time and then fleetly regulates CWSI in the canopy of plants [44, 45]. Second, H_2_O is insufficient to maintain its physiological activities when LWC is low in rice plants; the phenomenon quickly downregulates photosynthetic performance, such as declining *F*v/*F*m [46–48]. Third, SPAD biosynthesis would be hindered, and decomposition could be accelerated if LWC remains low for a long time, resulting in the yellowing of leaves [49]. Finally, the fluctuant sensitive parameters lead to significant changes in population characteristics, such as leaf area, biomass, and yield, presenting different degrees of decline [50]. Therefore, we deduce that CWSI, *F*v/*F*m, and SPAD should be introduced into the spectral monitoring model to improve the R^2^ for monitoring LWC in whole growth stages of rice plants.

### Coupling multi-source data for monitoring rice LWC

The canopy structure and growth environment of rice have been changing throughout the whole growth period, resulting in different spectral reflectance data, making it difficult to accurately build a water prediction model for the whole growth period with a single vegetation index model [51]. A good relationship between physiological and ecological indicators and spectral information is the key to rapid and non-destructive monitoring of coupled multi-source data. The relationships between CWSI, *F*v/*F*m, and SPAD and the slope (a) and intercept (b) of the conventional linear ND_(1287,1673)_ models were systematically analyzed at each observed period to achieve accurate prediction of LWC (Fig. 7). The parameter a had the closest relationship with CWSI with R^2^ of 0.92. However, the parameter b had the closest relationship with *F*v/*F*m with R^2^ of 0.99. These results indicate that the conventional linear ND_(1287,1673)_ models could be largely influenced by physiological and ecological factors when LWC was monitored using spectroscopic equipment. Therefore, slope and intercept could be defined as ecological and physiological factors in conventional linear ND_(1287,1673)_ models, especially for monitoring LWC in rice plants. This evidence confirms the necessity of incorporating physiological parameters into the conventional ND_(1287,1673)_ model to improve LWC monitoring accuracy in rice plants.

Currently, multi-source data are widely used in hyperspectral monitoring. However, most studies have utilized a single type of multi-source numbers to construct models independently or in combination, and few studies have discussed the effect of coupled multi-source data on the monitoring performance of rice LWC models. This study uses a commonly used multiple linear regression algorithm to estimate rice LWC based on coupled multi-source data and compare its monitoring performance. The results revealed that the monitoring capacities in ND_(1287,1673)_ models coupled with physiological and ecological factors were significantly increased than conventional ND_(1287,1673)_ models across all observed stages (Tables 3 and 4). The coupled model of ND + CWSI and ND + *F*v/*F*m had the best monitoring capacities at the booting to flowering and initial to middle grain filling stages, respectively. The different coupled parameters at different growth stages may related to the differences in rice growth characteristics. Previous studies have demonstrated that the canopy structure is unstable and that leaf area and biomass are in a state of constant growth during the booting and flowering stages in rice plants [52]. Irrigation regimes directly regulate canopy growth and development [53]. Therefore, the canopy micro-ecological factors, such as photosynthetically active radiation, temperature, and humidity, constantly fluctuate [54], especially for different water treatments. Finally, CWSI is a sensitive index at this stage because CWSI is mainly regulated by the micro-ecological factor [31, 32]. However, rice plants are in the senescence stage after flowering. Leaf photosynthetic capacity significantly declines during the grain-filling stage compared to pre-flowering in rice plants [43, 55]. Additionally, photosynthetic performances are susceptive to different irrigation regimes during senescence processes [56]. *F*v/*F*m is an important index evaluating photosynthetic performance. Therefore, photosynthetic performance could be a better multi-source parameter to predict LWC during the rice-filling stage.

### Comparison of machine learning algorithms

Machine learning algorithms have recently been widely used in model monitoring research and have become popular tools in precision agricultural production research. In this study, four machine learning algorithms, DT, RF, KNN, and GBDT were used to perform operations based on coupled models using rice flowering data as an example (Table 5). The results indicated that the R^2^ for the simulation and validation sets of DT, RF, KNN and GBDT were 0.80–0.93 and 0.75–0.86, respectively, which the R^2^ of machine learning algorithms were 1.16–1.35 times higher for the simulation sets and 1.09–1.25 times higher for the validation sets compared with MLR (Fig. 10). Among them, GBDT has the highest R^2^ with low and stable RMSE. Therefore, the GBDT model would be considered as the best machine learning algorithm for monitoring LWC in this study’s simulating and verification data sets. Previous research supports our results that the GBDT algorithm has higher prediction accuracy for leaf area in maize plants among the aforementioned algorithms [57]. GBDT can improve prediction accuracy by constructing a weak learner to correct the original model error via residuals and resultant iterations when the sample size is small [58]. The same weights are assigned for each input factor for the other algorithms; the algorithm would fail to determine the primary contributing factor in the input factor if the training data are insufficient [57]. This may be the primary reason the GBDT algorithm has significant advantages to improve LWC monitoring capabilities in this study because our sample size could not be abundant enough to support the data requirements of other algorithms. In summary, machine learning positively improves the monitoring abilities of LWC in rice plants.

### Future perspectives

Factors such as fertility period and rice LWC changes lead to parameter differences between relevant indicators extracted from various data sources. Current crop monitoring models are mostly single-factor statistical models that are difficult to consider crop growth and development, yield formation and its interactions with climate and soil environment, and lack universality and dynamics. Therefore, coupling spectral remote sensing information with multivariate data can construct a spectral monitoring model with high accuracy and stable reliability of crop growth, moisture content, and other indicators, providing an effective solution to the spectral monitoring problem. In this study, only four rice cultivars were considered after the booting stage, and the early growth of rice has yet to be monitored. Establishing a standardized water stress detection and diagnosis system based on water critical thresholds at various growth stages of rice will require the accumulation of data from multi-year, multi-point, continuous trials based on different rice variety types. This can accurately monitor the LWC to ensure high rice yield, improving water resource utilization efficiency and providing a basis for implementing precision agriculture.

## Conclusion

In this study, the LWC of rice with different cultivars, years, and water treatments was monitored based on multi-source data (physiological and ecological indicators and spectral information) with machine learning, explored the performance of single and combined multi-source data in LWC monitoring, and utilized different physiological indicators to establish two monitoring models for the growth differences of rice in the initial and middle stages. The results displayed that ND + CWSI had better monitoring performance in the early stage (booting to flowering stage), while ND + *F*v/*F*m was better in the late stage (initial to middle grain filling stage). Additionally, this study's newly constructed vegetation index ND_(1287,1673)_ also has good monitoring capacities for LWC in rice. Meanwhile, the machine learning algorithm (GBDT) further improves the monitoring performance of the model. In summary, this study confirms that using multi-source data and machine learning can improve the performance of hyperspectral prediction of rice LWC.

### Supplementary Information

**Additional file 1: Fig. S1.** The normal distribution characteristics of all parameters, linear distribution scatter plots and the corresponding correlation coefficient between LWC and physiological and ecological parameters including SPAD, *F*v/*F*m, *F*o, Y(II), CWSI, LWC, Biomass, and LAI at booting stage and Yield at maturity. **Fig. S2.** The normal distribution characteristics of all parameters, linear distribution scatter plots and the corresponding correlation coefficient between LWC and physiological and ecological parameters including SPAD, *F*v/*F*m, *F*o, Y(II), CWSI, LWC, Biomass, and LAI at flowering stage and Yield at maturity. **Fig. S3.** The normal distribution characteristics of all parameters, linear distribution scatter plots and the corresponding correlation coefficient between LWC and physiological and ecological parameters including SPAD, *F*v/*F*m, *F*o, Y(II), CWSI, LWC, Biomass, and LAI at initial grain filling stage and Yield at maturity. **Fig. S4.** The normal distribution characteristics of all parameters, linear distribution scatter plots and the corresponding correlation coefficient between LWC and physiological and ecological parameters including SPAD, *F*v/*F*m, *F*o, Y(II), CWSI, LWC, Biomass, and LAI at middle grain filling stage and Yield at maturity. **Table S1.** The maximum, minimum, and mean values of the main measured parameters and standard deviation and coefficient of variation of each parameter in this study. **Table S2.** The multicollinearity test between ND and SPAD, between ND and Fv/Fm, between ND and CWSI at different observed periods based on the tolerance and variance inflation factor values and Durbin Watson test of multivariate linear regressed models presented in Table 4 in text.

## Data Availability

The datasets used and/or analyzed during the current study are available from the corresponding author on reasonable request.
