# Supplementary material for: Improving the prediction performance of leaf water content by coupling multi-source data with machine learning in rice (Oryza sativa L.)
Source: Plant Methods. 2024 Mar 23;20:48. doi: 10.1186/s13007-024-01168-5 (PMC10960999; doi:10.1186/s13007-024-01168-5)
Supplement: Supplementary file 1 — Additional file 1: Fig. S1. The normal distribution characteristics of all parameters, linear distribution scatter plots and the corresponding correlation coefficient between LWC and physiological and ecological parameters including SPAD, Fv/Fm, Fo, Y(II), CWSI, LWC, Biomass, and LAI at booting stage and Yield at maturity. Fig. S2. The normal distribution characteristics of all parameters, linear distribution scatter plots and the corresponding correlation coefficient between LWC and physiological and ecological parameters including SPAD, Fv/Fm, Fo, Y(II), CWSI, LWC, Biomass, and LAI at flowering stage and Yield at maturity. Fig. S3. The normal distribution characteristics of all parameters, linear distribution scatter plots and the corresponding correlation coefficient between LWC and physiological and ecological parameters including SPAD, Fv/Fm, Fo, Y(II), CWSI, LWC, Biomass, and LAI at initial grain filling stage and Yield at maturity. Fig. S4. The normal distribution characteristics of all parameters, linear distribution scatter plots and the corresponding correlation coefficient between LWC and physiological and ecological parameters including SPAD, Fv/Fm, Fo, Y(II), CWSI, LWC, Biomass, and LAI at middle grain filling stage and Yield at maturity. Table S1. The maximum, minimum, and mean values of the main measured parameters and standard deviation and coefficient of variation of each parameter in this study. Table S2. The multicollinearity test between ND and SPAD, between ND and Fv/Fm, between ND and CWSI at different observed periods based on the tolerance and variance inflation factor values and Durbin Watson test of multivariate linear regressed models presented in Table 4 in text. [file 13007_2024_1168_MOESM1_ESM.doc]

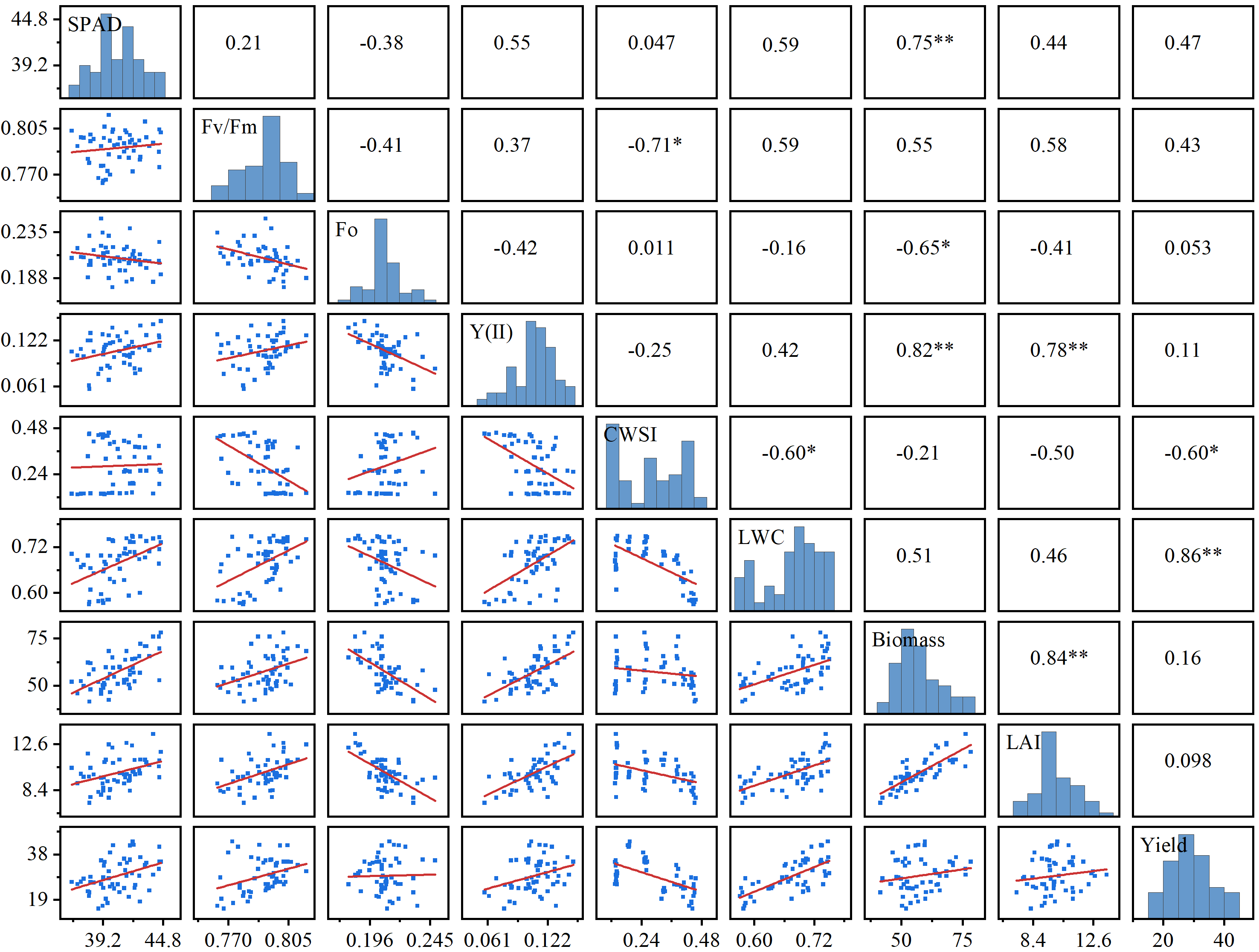


Fig. S1. The normal distribution characteristics of all parameters, linear distribution scatter plots and the corresponding correlation coefficient between LWC and physiological and ecological parameters including SPAD, *F*v/*F*m, *F*o, Y(Ⅱ), CWSI, LWC, Biomass, and LAI at booting stage and Yield at maturity. SPAD: chlorophyll content; *F*v/*F*m: maximum photochemical efficiency; *F*o: minimal fluorescence; Y(Ⅱ): actual photochemical efficiency; CWSI: crop water stress index; LWC: leaf water content; Biomass: above-ground biomass; LAI: leaf area index; * and ** indicate significant correlation at 5% and 1% probability level, respectively.


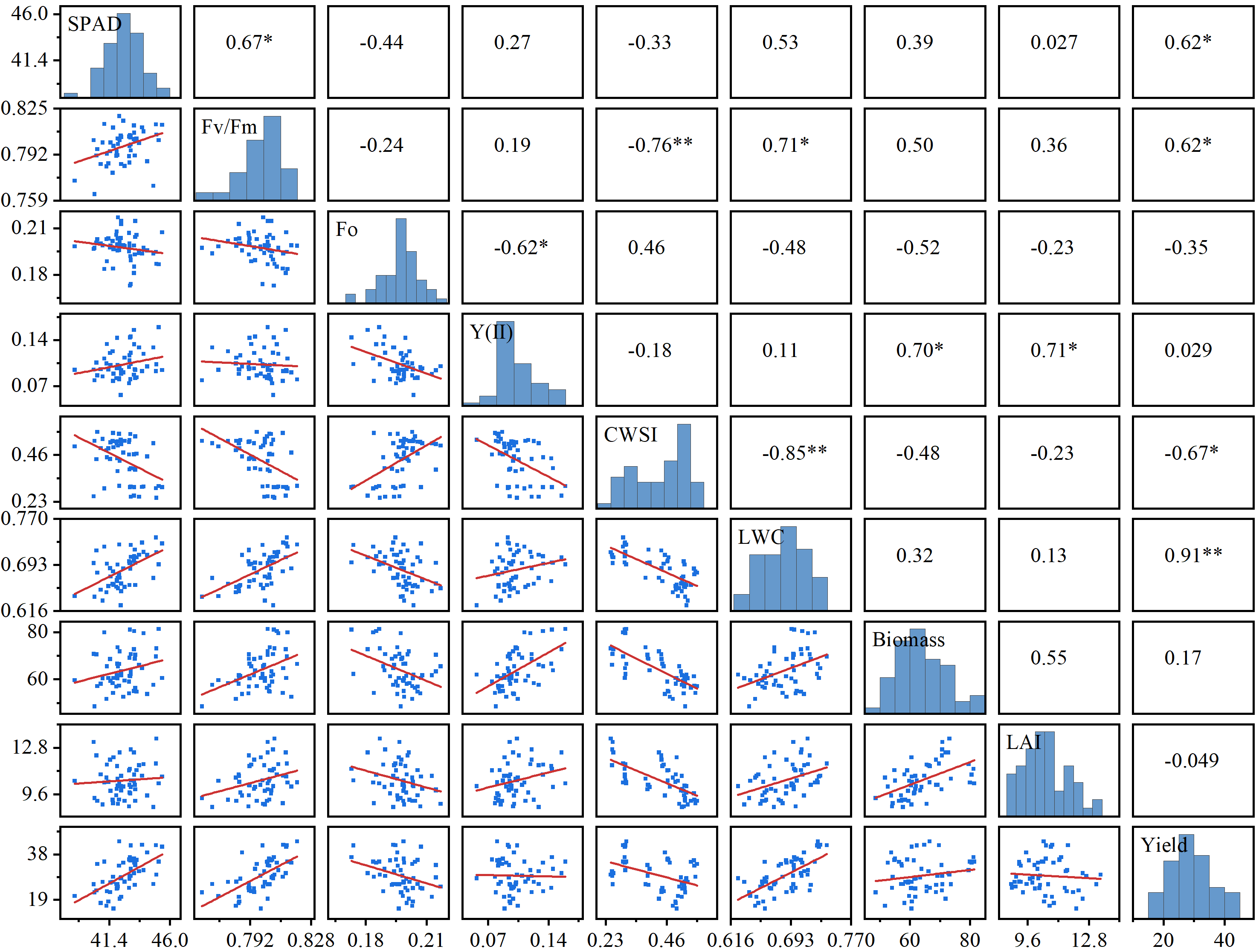


Fig. S2. The normal distribution characteristics of all parameters, linear distribution scatter plots and the corresponding correlation coefficient between LWC and physiological and ecological parameters including SPAD, *F*v/*F*m, *F*o, Y(Ⅱ), CWSI, LWC, Biomass, and LAI at flowering stage and Yield at maturity. * and ** indicate significant correlation at 5% and 1% probability level, respectively.


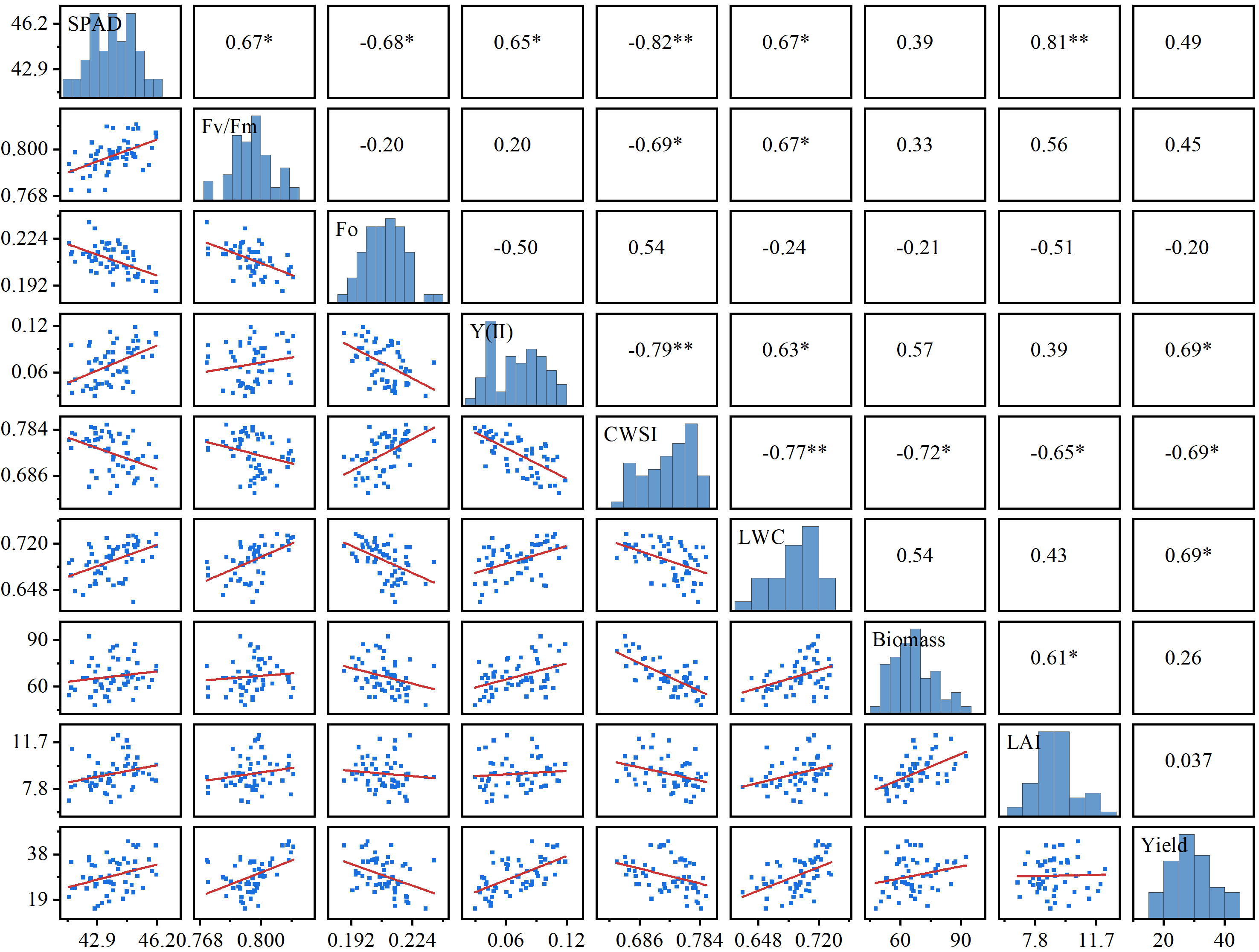


Fig. S3. The normal distribution characteristics of all parameters, linear distribution scatter plots and the corresponding correlation coefficient between LWC and physiological and ecological parameters including SPAD, *F*v/*F*m, *F*o, Y(Ⅱ), CWSI, LWC, Biomass, and LAI at initial grain filling stage and Yield at maturity * and ** indicate significant correlation at 5% and 1% probability level, respectively.


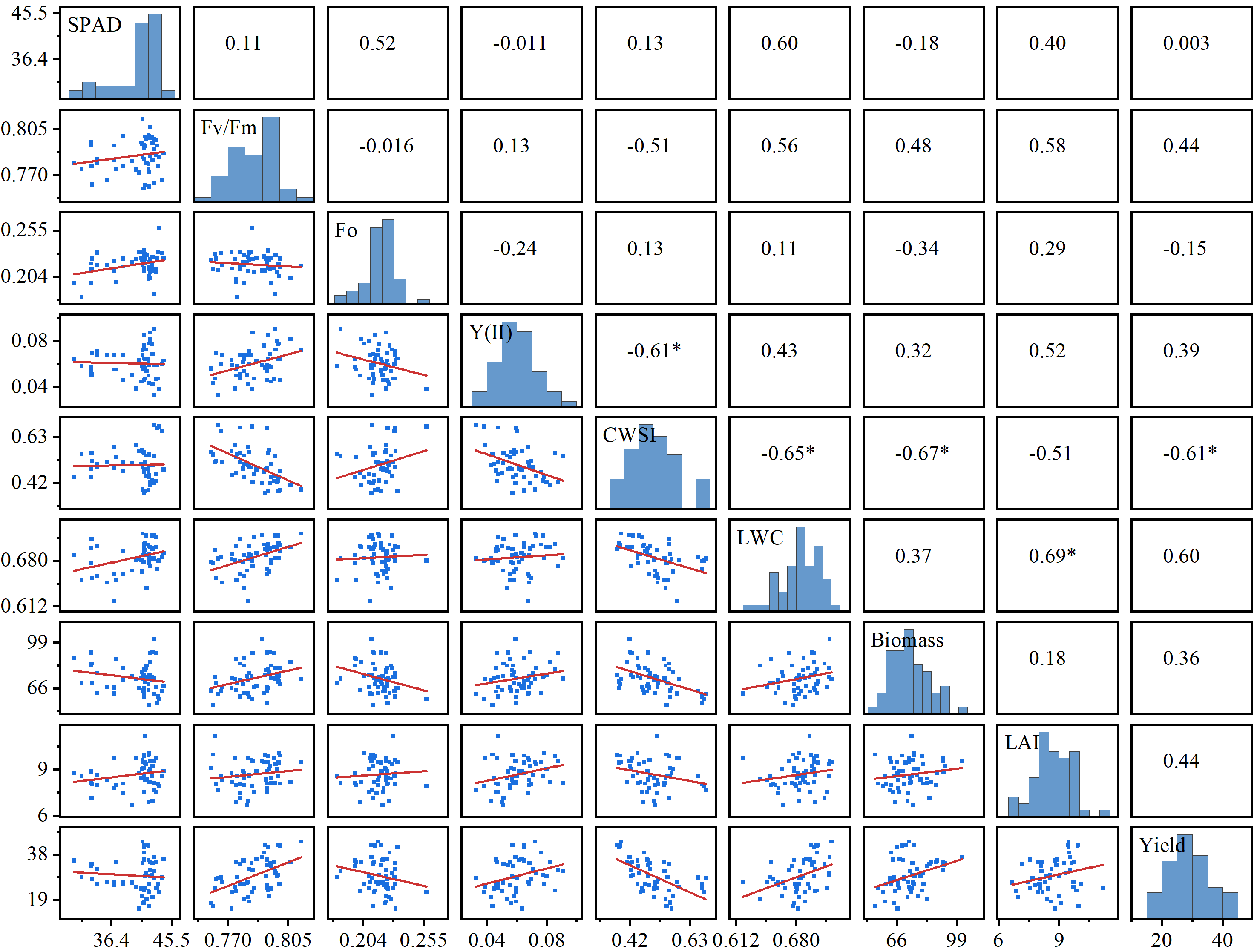


Fig. S4. The normal distribution characteristics of all parameters, linear distribution scatter plots and the corresponding correlation coefficient between LWC and physiological and ecological parameters including SPAD, *F*v/*F*m, *F*o, Y(Ⅱ), CWSI, LWC, Biomass, and LAI at middle grain filling stage and Yield at maturity * and ** indicate significant correlation at 5% and 1% probability level, respectively.

| Table S1. The maximum, mimnimum, and mean values of the main measured parameters and standard deviation and coefficient of variation of each parameter in this study. | | | | | | |
| --- | --- | --- | --- | --- | --- | --- |
| Growth stage | Parameters | Max | Min | Mean | Standard deviation | Coefficient of variation |
| Booting | ND | 0.12 | 0.08 | 0.10 | 0.01 | 0.11 |
|  | SPAD | 44.56 | 36.26 | 40.78 | 1.90 | 0.05 |
|  | Fv/Fm | 0.81 | 0.76 | 0.78 | 0.01 | 0.01 |
|  | CWSI | 0.45 | 0.13 | 0.30 | 0.11 | 0.36 |
|  | LWC | 0.75 | 0.57 | 0.67 | 0.05 | 0.07 |
| Flowering | ND | 0.11 | 0.09 | 0.10 | 0.01 | 0.08 |
|  | SPAD | 45.40 | 38.80 | 42.60 | 1.29 | 0.03 |
|  | Fv/Fm | 0.82 | 0.76 | 0.80 | 0.01 | 0.01 |
|  | CWSI | 0.57 | 0.24 | 0.42 | 0.09 | 0.21 |
|  | LWC | 0.73 | 0.62 | 0.69 | 0.03 | 0.04 |
| Initial grain filling | ND | 0.12 | 0.08 | 0.10 | 0.01 | 0.09 |
|  | SPAD | 46.16 | 41.33 | 43.65 | 1.15 | 0.03 |
|  | Fv/Fm | 0.81 | 0.77 | 0.79 | 0.01 | 0.01 |
|  | CWSI | 0.79 | 0.64 | 0.73 | 0.04 | 0.05 |
|  | LWC | 0.73 | 0.62 | 0.69 | 0.02 | 0.03 |
| Middle grain filling | ND | 0.12 | 0.07 | 0.10 | 0.01 | 0.12 |
|  | SPAD | 44.27 | 30.70 | 40.16 | 3.52 | 0.09 |
|  | Fv/Fm | 0.81 | 0.76 | 0.78 | 0.01 | 0.02 |
|  | CWSI | 0.68 | 0.37 | 0.51 | 0.08 | 0.15 |
|  | LWC | 0.72 | 0.62 | 0.68 | 0.02 | 0.03 |

| Table S2. The multicollinearity test between ND and SPAD, between ND and Fv/Fm, between ND and CWSI at different observed perids based on the tolerance and variance inflation factor values and Durbin Watson test of multivariate linear regressed models presented in Table 4 in text. | | | | | | | | | | | |
| --- | --- | --- | --- | --- | --- | --- | --- | --- | --- | --- | --- |
| Growth stage | ND+SPAD | | | | ND+Fv/Fm | | | | ND+CWSI | | |
|  | Durbin watson | Tolerance | Variance inflation factor | Durbin watson | | Tolerance | Variance inflation factor | Durbin watson | | Tolerance | Variance inflation factor |
| Booting | 1.03 | 0.90 | 1.11 | 1.45 | | 0.98 | 1.02 | 0.76 | | 0.73 | 1.37 |
| Flowering | 1.40 | 0.74 | 1.35 | 1.82 | | 0.76 | 1.31 | 1.47 | | 0.24 | 4.20 |
| Initial grain filling | 2.03 | 0.72 | 1.40 | 2.04 | | 0.60 | 1.67 | 1.95 | | 0.51 | 1.95 |
| Middle grain filling | 1.79 | 0.76 | 1.31 | 1.87 | | 0.73 | 1.38 | 1.71 | | 0.72 | 1.39 |
